# Impact of SARS-CoV-2 Vaccination on Disease Activity and Severity of COVID-19 Infection in Patients with Systemic Lupus Erythematosus: A Multicenter Cohort Study

**DOI:** 10.3390/vaccines13101074

**Published:** 2025-10-21

**Authors:** Natália Sarzi Sartori, Ketty Lysie Libardi Lira Machado, Samira Tatiyama Miyamoto, Flavia Zon Pretti, Maria da Penha Gomes Gouveia, Yasmin Gurtler Pinheiro de Oliveira, Vanezia Gonçalves da Silva, Filipe Faé, Ana Paula Neves Burian, Karina Rosemarie Lallemand Tapia, Anna Carolina Simões Moulin, Luiza Lorenzoni Grillo, Paula dos Santos Athayde, Helena da Silva Corona, Sabrina de Souza Ramos, Flávia Maria Matos Melo Campos Peixoto, Priscila Dias Cardoso Ribeiro, Vanessa de Oliveira Magalhães, Mariana Freitas de Aguiar, Erika Biegelmeyer, Cristiane Kayser, Alexandre Wagner Silva De Souza, Charlles Heldan de Moura Castro, Juliana Bühring, Sandra Lúcia Euzébio Ribeiro, Sérgio Henrique Oliveira dos Santos, Clara Pinheiro Martins, Jonathan Willian da Silva Rodrigues, Marcos Mavignier Sousa Dias, Bruna Guimarães Dutra, Camila Maria Paiva França Telles, Samuel Elias Basualto Dias, Rodrigo Poubel Vieira de Rezende, Katia Lino Baptista, Rodrigo Cutrim Gaudio, Ana Karla Guedes de Melo, Valéria Bezerra da Silva, Vitor Alves Cruz, Jozelia Rêgo, Rejane Maria Rodrigues de Abreu Vieira, Adah Sophia Rodrigues Vieira, Adriana Maria Kakehasi, Anna Carolina Faria Moreira Gomes Tavares, Artur José Azevedo Pereira, Pollyana Vitoria Thomaz da Costa, Valderilio Feijó Azevedo, Nicole Pamplona Bueno de Andrade, Guilherme Levi Tres, Olindo Assis Martins-Filho, Vanessa Peruhype-Magalhães, Valéria Valim, Gilda Aparecida Ferreira, Andréa Teixeira-Carvalho, Edgard Torres dos Reis-Neto, Emilia Inoue Sato, Marcelo de Medeiros Pinheiro, Viviane Angelina de Souza, Ricardo Machado Xavier, Gecilmara Salviato Pileggi, Odirlei André Monticielo

**Affiliations:** 1 Serviço de Reumatologia, Hospital de Clínicas de Porto Alegre, Universidade Federal do Rio Grande do Sul (UFRGS), Porto Alegre 90010-150, RS, Brazil; 2 Hospital Universitário Cassiano Antônio Moraes (HUCAM), Universidade Federal do Espírito Santo (UFES), Vitória 29041-295, ES, Brazil; 3 Centro de Referências para Imunobiológicos Especiais (CRIE) da Secretaria de Saúde do Estado do Espírito Santo, Vitória 29041-295, ES, Brazil; 4 Escola Paulista de Medicina (EPM), Universidade Federal de São Paulo (UNIFESP), São Paulo 04023-062, SP, Brazil; 5 Department of Rheumatology, Universidade Federal do Amazonas (UFAM), Manaus 69080-900, AM, Brazil; 6 Department of Rheumatology, Universidade Federal Fluminense (UFF), Niterói 24220-900, RJ, Brazil; 7 Hospital Universitário Lauro Wanderley, Universidade Federal da Paraíba (UFPB), João Pessoa 58051-900, PB, Brazil; 8 Department of Rheumatology, Universidade Federal de Goiás (UFG), Goiânia 74690-900, GO, Brazil; 9 Department of Rheumatology, Universidade de Fortaleza (UNIFOR), Fortaleza 60811-905, CE, Brazil; 10 Hospital das Clínicas da Universidade Federal de Minas Gerais, Belo Horizonte 31270-901, MG, Brazil; 11 Department of Rheumatology, Universidade Federal de Juiz de Fora (UFJF), Juiz de Fora 36036-900, MG, Brazil; 12 Edumed—Educação em Saúde S/S Ltda, Curitiba 80440-080, PR, Brazil; 13 Instituto Renè Rachou, Fundação Oswaldo Cruz (FIOCRUZ-Minas), Belo Horizonte 30190-002, MG, Brazil; 14 Locomotor System Department, Faculdade de Medicina, Universidade Federal de Minas Gerais (UFMG), Belo Horizonte 30130-100, MG, Brazil; 15 Faculdade de Medicina, Universidade Federal de Juiz de Fora, Juiz de Fora 36036-900, MG, Brazil

**Keywords:** COVID-19 vaccination, vaccination against SARS-CoV-2, safety, systemic lupus erythematosus

## Abstract

Background: To prospectively evaluate the safety and clinical impact of SARS-CoV-2 vaccines in patients with systemic lupus erythematosus (SLE). Methods: Subanalysis of the Brazilian multicenter observational study “Safety, Effectiveness and Duration of Immunity after Vaccination against SARS-CoV-2 in Patients with Immune-Mediated Inflammatory Diseases (SAFER)”, which included SLE patients vaccinated with CoronaVac, ChAdOx1, or BNT162b2. Patients with HIV infection, pregnant women, or those with immunosuppression not related to SLE were excluded. Safety data related to adverse events and underlying disease activity were assessed. Additionally, COVID-19 cases were monitored throughout the follow-up period. Results: The study included 373 patients with systemic lupus erythematosus (SLE), with a mean age of 36 years, the majority being women (89.8%). The most common adverse events after SARS-CoV-2 vaccination were injection site reactions and headache, observed both after the first and subsequent doses. The ChAdOx-1 vaccine was associated with a higher frequency of adverse events compared to CoronaVac. At baseline, 38.3% of patients were in remission, 32.8% had low disease activity, and 28.9% had moderate to high activity. Following CoronaVac vaccination, there was an increase in remission rates (from 34.6% to 51.1%) and a significant reduction in moderate to high activity (from 37.6% to 15.0%) after the first dose, with this reduction partially maintained after the second dose. In contrast, patients vaccinated with ChAdOx-1 showed an increase in moderate to high activity (from 14.5% to 38.2% after the first dose), a trend that persisted after the second dose. No statistically significant changes in disease activity were observed among those who received BNT162b2. During follow-up, 44 cases of COVID-19 were reported, all mild, with no deaths or need for intensive care unit admission. Conclusions: Vaccination against SARS-CoV-2 demonstrated a favorable safety profile in patients with SLE, with a low frequency of serious adverse events. While analysis of disease activity revealed variations across vaccine platforms, most notably an increased proportion of moderate to high disease activity among those receiving ChAdOx-1 compared with CoronaVac and BNT162b2, the overall occurrence of COVID-19 during follow-up was limited to mild cases, with no severe outcomes. These findings highlight that, despite potential risks of disease exacerbation, the clear protection against severe COVID-19 supports vaccination as a beneficial strategy for this immunocompromised population.

## 1. Introduction

The prevention of infectious and contagious diseases through vaccination is one of the most impactful public health measures for controlling the spread of harmful microorganisms. The history of vaccines is closely linked to the development of the smallpox vaccine, and vaccination is the reason why many diseases are now eradicated or under control [1]. The COVID-19 pandemic has reaffirmed the importance of infection prevention through vaccination, aiming to reduce the likelihood of severe outcomes from SARS-CoV-2 infection in the general population, and particularly in individuals at high risk, such as patients with immune-mediated rheumatic diseases [2].

Systemic lupus erythematosus (SLE), like other immune-mediated diseases, carries a higher risk of developing infections due to immunological factors associated with the underlying disease and the immunosuppressive therapy used to control disease activity [3]. In this context, infection prevention strategies, such as vaccination, are essential for the proper management and reduction of infections in these patients. Recommendations emphasizing this approach have been published with the aim of guiding and encouraging vaccination in this population [4,5,6,7].

On the other hand, although vaccination is recommended and should be considered on an individual basis for each patient, low adherence is observed among individuals with SLE [8,9]. This hesitancy, present in both patients and physicians, stems from concerns about safety, particularly regarding the risk of disease exacerbation, as immunization induces activation of the immune system [10]. As a result, lower vaccination coverage rates are observed for various vaccines in these patients compared to the general population [11,12].

Vaccines against SARS-CoV-2 were rapidly introduced to the market through an accelerated development process, due to the emergency context of the COVID-19 pandemic. In this context, uncertainties were raised about the safety profile, especially in patients with systemic lupus erythematosus (SLE), due to the potential risk of worsening autoimmune phenomena [13,14]. Postmarketing surveillance has played a crucial role in this scenario, especially as, beyond the initial vaccination, the administration of booster doses has been recommended.

Post-vaccination seroconversion in patients with immune-mediated diseases has been demonstrated in previous studies, with lower rates compared to the general population, though still reaching antibody titers considered protective against COVID-19 infection in the short term [15]. However, most studies assessed immunogenicity without medium- to long-term follow-up after vaccination, and data on the clinical effectiveness of SARS-CoV-2 vaccines in patients with systemic lupus erythematosus (SLE) remain scarce. In particular, there is a lack of long-term, real-world evidence directly comparing the effects of different COVID-19 vaccine platforms in this population. In this context, we conducted this real-world study with extended follow-up to assess postmarketing safety and the clinical effectiveness of COVID-19 vaccines in patients with SLE.

## 2. Materials and Methods

### 2.1. Study Design and Participants

This study analyzed a subgroup of patients with systemic lupus erythematosus (SLE) from the multicenter cohort of the SAFER study—Safety, Effectiveness, and Duration of Immunity After SARS-CoV-2 Vaccination in Patients with Immune-Mediated Inflammatory Diseases, a prospective Brazilian study that began in June 2021. Patients included in this analysis were those with SLE who received active immunization against SARS-CoV-2. Eligibility criteria for the study were: age over 18 years, fulfillment of the 2019 American College of Rheumatology/European League Against Rheumatism (ACR/EULAR) classification criteria for SLE, and indication for COVID-19 vaccination. Exclusion criteria included a history of severe hypersensitivity reaction to vaccines, pregnancy, malignancy (except in situ cervical cancer and non-melanoma skin cancer), and HIV infection.

Three vaccines were evaluated: CoronaVac (Inactivated SARS-CoV-2 Vaccine, Beijing, China and Instituto Butantan, São Paulo, Brazil), ChAdOx1 (AstraZeneca, Cambridge, UK), and BNT162b2 (Pfizer—BioNTech, Mainz, Germany). All individuals included in the analysis received at least the primary vaccination series, consisting of two doses. The administration of these vaccines was supervised, but the choice of vaccine was determined by the attending physician, preserving the observational nature of the study.

### 2.2. Outcomes and Variables

Epidemiological data, as well as information on comorbidities, disease duration, and prior vaccination history, were collected during the baseline assessment.

Patients were evaluated both in person and via telephone contact. All participants attended an in-person visit at the time of study enrollment (T1). Subsequent assessments were conducted at the following time points: 28 days after the first dose (T2), 28 days after the second dose (T3), 28 days after the third dose (T4), and finally at two additional time points depending on the vaccination schedule received. For patients who did not receive a third dose, an evaluation was performed 12 months after the second dose. For those who completed the vaccination schedule with a third dose, the final assessment occurred between 4 and 6 months after the additional dose. Post-vaccination event surveillance was also carried out through symptom recording by the participant in a printed diary provided at the time of vaccine administration.

Disease activity was assessed using the SLEDAI-2K score, categorized as remission (SLEDAI-2K = 0), low activity (SLEDAI-2K 1–5), and moderate to high activity (SLEDAI-2K ≥ 6). This score was recorded at baseline and at subsequent visits to evaluate the safety outcome related to SLE exacerbation. Moderate exacerbation was defined as an increase of 3 or more points in the SLEDAI-2K score, while severe exacerbation was defined as an increase greater than 12 points in the SLEDAI-2K.

Regarding clinical effectiveness, we collected data on the number of confirmed COVID-19 cases, hospitalizations, intensive care unit (ICU) admissions, and deaths secondary to SARS-CoV-2 infection. Surveillance of symptomatic COVID-19 cases was conducted remotely, either periodically every two weeks or reactively. COVID-19 cases were confirmed through laboratory testing or defined based on clinical-epidemiological diagnosis (i.e., clinical symptoms of flu-like syndrome and household contact with an individual diagnosed with COVID-19) [16,17].

Biological samples were collected during visits before and after vaccination and included laboratory parameters and serology (IgG) for SARS-CoV-2, performed by chemiluminescence using the Elecsys^®^ Anti-SARS-CoV-2 S immunoassay (Roche, Rotkreuz, Switzerland). An assessment of the humoral immune response to SARS-CoV-2 vaccination was also conducted in patients without prior COVID-19 infection, comparing seroconversion rates and IgG antibody titers between those who developed COVID-19 during follow-up and those who remained uninfected. The other immunogenicity findings have been reported in a prior publication by the multicenter cohort of the SAFER study [18].

All data presented were stored in a centralized electronic database (REDCap—Research Electronic Data Capture (Redcap—Research Electronic Data Capture, https://redcap.reumatologia.org.br/, accessed on 14 October 2025).

### 2.3. Statistical Analysis

Analyses were performed using Stata (v.17) and R (v.4.2.0) software. A statistical significance level of 5% and a 95% confidence interval were used for all tests.

A descriptive analysis was conducted on demographic and epidemiological characteristics, as well as on reactogenicity following each dose of the vaccines.

Proportions between groups were compared using the chi-square test and Fisher’s exact test for categorical variables. Mean and standard deviation (SD), as well as median and interquartile range (IQR), were calculated for continuous variables and analyzed by ANOVA and Wilcoxon (2 groups) or Kruskal–Wallis (>2 groups), respectively. Disease activity before and after vaccination was compared using the McNemar test. For the analysis of IgG titers, data normalization was performed using the base-10 logarithm. The analysis of normalized IgG titers and SLEDAI scores over time was conducted using the non-parametric Wilcoxon/Mann–Whitney test with Bonferroni correction.

Statistical analyses were conducted using Stata version 17 and R version 4.2.0 software packages.

### 2.4. Ethical Aspects

This research (CAAE 43479221.0.1001.5505) was duly reviewed and approved by the Research Ethics Committee of the coordinating center, Federal University of São Paulo, as well as by the Ethics Committees of the other participating centers. All participants signed and dated the Informed Consent Form, in accordance with the ethical guidelines governing research involving human subjects.

## 3. Results

**Figure 1:**
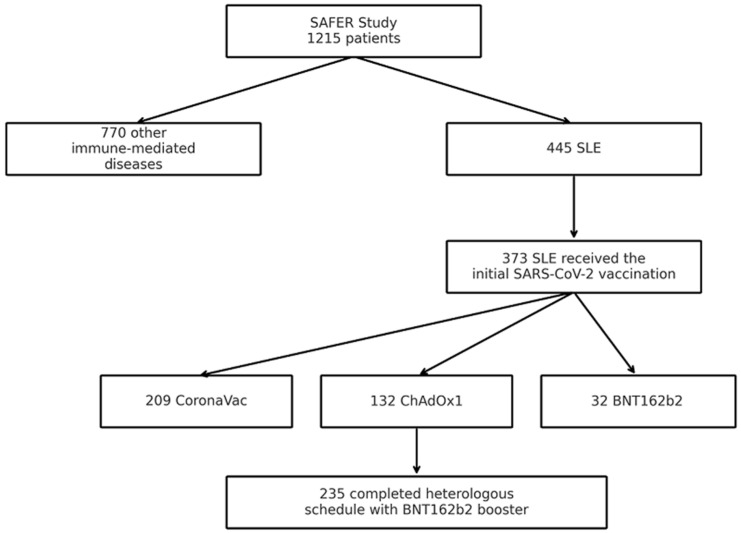
Among the 1215 participants, 445 had a diagnosis of systemic lupus erythematosus (SLE). Of these, 373 followed the evaluated COVID-19 vaccination schedules and were included in the analysis. Among them, 235 patients subsequently received a heterologous booster dose with BNT162b2, completing the three-dose regimen.

Our cohort included 1215 patients; for this study, a total of 373 patients with systemic lupus erythematosus (SLE) who received the initial SARS-CoV-2 vaccination schedule were evaluated. Among these patients, 209 received the initial vaccination schedule with two doses of CoronaVac, 132 received two doses of ChAdOx1, and 32 received BNT162b2 as the initial immunization regimen. Two hundred and thirty-five patients completed a heterologous COVID-19 vaccination schedule, consisting of the initial two-dose series followed by a third booster dose with the BNT162b2 vaccine (Figure 1).

Among these patients, the majority were women, totaling 335 (89.81%). The mean age of these patients was 36 years (SD; 28–45 years). Regarding skin color, 196 (52.55%) self-identified as mixed, 114 (30.56%) as white, 54 (14.48%) as black, 8 (2.14%) as Asian, and 1 (0.27%) as Indigenous. The median disease duration of SLE in this cohort was 9 years (IQR; 4–15 years). Regarding past medical history, 156 (41.82%) patients had no comorbidities, 107 (28.69%) had systemic arterial hypertension, 47 (12.50%) had a history of thrombotic events regardless of an antiphospholipid syndrome (APS) diagnosis, and 30 (8.09%) had an APS diagnosis.

Considering post-vaccination adverse events, symptoms at the injection site (local pain), followed by headache e fatigue, were the most commonly reported events by patients after both the first and subsequent doses. Among the different vaccine platforms, a statistically significant difference was observed after the first dose, with more adverse events reported in patients who received the ChAdOx1 vaccine compared to those who received the inactivated virus vaccine. After the second dose, this statistically significant trend persisted only for local symptoms such as induration and pain, with a higher number of events observed in patients who received the ChAdOx1 vaccine. Among patients who completed the full three-dose vaccination schedule, no statistically significant differences were observed between the groups that received the third dose with the BNT162b2 vaccine, except for the adverse event of local hematoma, which was more frequently reported among those who had previously received the ChAdOx1 vaccine (Table A1).

In relation to disease activity and its frequency, at inclusion, 138 (38.33%) patients were in disease remission, 118 (32.78%) patients had low disease activity, and 104 (28.89%) patients had moderate to high disease activity. At the assessment 28 days after the first dose, 105 (48.61%) were in remission, 60 (27.78%) had low activity, and 51 (23.61%) had moderate to high disease activity. At the evaluation 28 days after the second dose of the vaccine, 146 (45.06%) were in disease remission, 96 (29.63%) had low disease activity, and 82 (25.31%) had moderate to high disease activity. This distribution of disease activity levels among the different vaccine platforms was statistically significant (*p* < 0.01) in the evaluation 28 days after the first dose, with a higher frequency of individuals in moderate to high disease activity in the ChadOx-1 group compared to the CoronaVac group (Table 1).

We conducted a paired analysis of disease activity by vaccine platform over time, after the administration of the vaccine doses. With the use of CoronaVac, we observed that after the first dose, there was a significant increase in the proportion of individuals in remission (*p* = 0.002) and a statistically significant decrease in the number of individuals with moderate to high disease activity (*p* < 0.001). After the second dose, there were no significant changes in the number of individuals in remission or with low disease activity. However, there was a statistically significant reduction in the number of individuals with moderate to high disease activity (*p* = 0.004). When evaluating the group of individuals who received the ChadOx-1 vaccine regimen, after the first dose, there were no significant changes in the number of individuals in remission. However, there was a decrease in the proportion of individuals with low disease activity and an increase in the proportion of individuals with moderate to high disease activity, both with statistically significant differences (*p* = 0.004 and *p* = 0.001, respectively). After the second dose, the same trend was observed, there were no significant changes in the number of individuals in remission, and there was a significant decrease in the number of individuals with low disease activity (*p* = 0.011) and a significant increase in the number of individuals classified as having moderate to high disease activity (*p* = 0.028). Regarding individuals who received the BNT162b2 regimen, after the second dose, there were no significant changes in the number of individuals in remission, with low activity, or with moderate to high disease activity. Data related to disease activity 28 days after the first dose of BNT162b2 was recorded for only one patient, who was in low disease activity (Table 2).

In the analysis of exacerbation over time, it was observed that, after the administration of each vaccine dose, 80.0% of the patients did not present disease exacerbation. Evaluating the different vaccination regimens at each dose of the immunizing agent, after the first vaccine dose, the group that received the CoronaVac vaccine had a lower incidence of moderate flare (8.27%) and severe flare (0.75%) when compared to the patients who received the ChadOx-1 vaccine, in which a higher incidence of moderate flare (31.58%) and severe flare (7.89%) was observed, *p* < 0.001. In the assessment conducted 28 days after the second dose, the ChadOx-1 vaccine showed a higher incidence of moderate flare (26.27%) and severe flare (5.08%) compared to CoronaVac (15.79% and 0.58%, respectively) and BNT162b2 (3.23% for both types of flare), *p* < 0.001. Considering the analysis 28 days after the complete vaccination regimen (two initial doses plus the booster dose with BNT162b2), flares were also more frequent in the groups that received ChadOx-1 (28.57% moderate; 2.60% severe) and BNT162b2 (42.86% moderate; 0.00% severe), compared to the CoronaVac group (10.08% moderate; 0.00% severe), *p* = 0.001 (Table 3).

In patients with the presence of exacerbation, we evaluated the manifestations according to the SLEDAI-2K domain considering subgroups with moderate and severe exacerbation. We found after the first dose, among patients with moderate variation in SLEDAI (*n* = 35; total *n* evaluated 210), a marked increase in musculoskeletal manifestations (*n* = 21), followed by cutaneous manifestations and the presence of proteinuria, and among patients with severe exacerbation (*n* = 7; total *n* evaluated 210), presence of lupus headache, visual alteration, and proteinuria. After the second dose, of the patients with moderate exacerbation (*n* = 59; total *n* 320), a predominance of articular and cutaneous manifestations was observed, and some patients with presence of proteinuria and lupus headache; among patients with severe flare (*n* = 8; total *n* 320), lupus headache, arthritis, cutaneous rash, visual alteration, and proteinuria were the most commonly presented manifestations. In the patients evaluated who completed a 3-dose vaccination schedule, patients with moderate exacerbation (*n* = 40, total *n* evaluated 210), the main manifestations presented were also arthritis and cutaneous symptoms, followed by the presence of leukocyturia and proteinuria. After the third dose, severe flare occurred only in 2 patients, with the main manifestations being headache and visual alteration (Table A2).

We also followed the outcome of these patients regarding COVID-19 infection after immunization. Of the total 373 patients included, 9 cases of COVID-19 were registered up to 15 days after the first dose of the vaccine. We identified 44 cases counted from 15 days after the second dose up to 12 months after the second dose or the period immediately before the administration of the fourth dose of the vaccine. Among the patients with infection, eight patients sought medical care in an emergency unit and only one patient required hospital admission. Admission to an intensive care unit or deaths were not recorded. There was no statistically significant difference in the number of new COVID-19 cases among the different vaccine platforms. Twenty-five cases (11.96%) occurred in patients who completed the initial schedule with CoronaVac, 18 cases (13.64%) in patients who completed the initial schedule with the ChadOx-1 vaccine, and 1 case (3.13%) in patients with the BNT162b2 vaccine schedule, with a *p*-value of 0.26.

**Figure 2:**
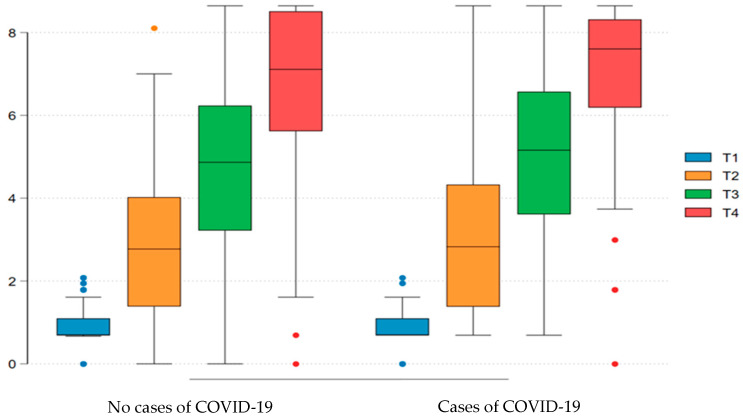
Humoral response (IgG titers expressed in log10) after vaccination considering only cases with non-reactive serology at Inclusion: comparison of patients with COVID-19 versus those without a diagnosis of COVID-19 at follow-up.

We performed a subanalysis of immunogenicity in patients without a prior clinical history of COVID-19 at baseline, comparing those who developed COVID-19 infection during the follow-up period with those who remained uninfected. Serology performed 28 days after the first dose of the SARS-CoV-2 vaccine was reactive in 92/156 (58.97%) of patients without COVID-19 and in 26/44 (59.09%) of those who subsequently developed COVID-19, with no statistically significant difference observed. Following the second dose, seropositivity rates increased to 126/151 (83.44%) in the uninfected group and 38/43 (88.37%) in the infected group (*p* > 0.05). After the third dose, seropositivity was observed in 130/136 (95.59%) of patients without infection and 34/37 (91.89%) of those with infection, again without significant difference (*p* > 0.05). Assessment of the humoral response based on IgG titers expressed as Log10 revealed a median value 28 days after the first dose of 2.77 (IQR: 1.39–4.03) in the uninfected group and 2.83 (IQR: 1.38–4.33) in the infected group (*p* = 0.52). IgG titers 28 days after the second and third doses were 4.87 (IQR: 3.22–6.23) and 7.11 (IQR: 5.62–8.51) in the uninfected group, compared to 5.16 (IQR: 3.61–6.57) and 7.60 (IQR: 6.19–8.32) in the infected group, with no statistically significant differences observed between groups (Figure 2).

Humoral response to COVID-19 vaccination, assessed by IgG titers (on a log10 scale), in patients with negative serology at inclusion. The comparison was made between individuals who did not present COVID-19 infection during follow-up and those who had confirmed infection. Titers were assessed at four time points (T1 to T4). A progressive increase in the humoral response was observed between the groups, with no statistically significant difference. T1: inclusion visit; T2: visit 28 days after the first dose of vaccine; T3: visit 28 days after the second dose of vaccine; T4: visit 28 days after the third dose of vaccine (complete vaccination schedule with 3 doses or heterologous with the 3rd dose of BNT162b2 vaccine).

## 4. Discussion

Our study aimed to evaluate the safety of SARS-CoV-2 vaccines in patients with systemic lupus erythematosus (SLE) through a prospective multicenter study, employing an active surveillance plan for adverse events associated with vaccination. We reported the presence of vaccine-related adverse events in these individuals both after the first dose and subsequent doses; however, non-severe events were more frequent, primarily related to symptoms at the injection site, followed by systemic symptoms such as headache, musculoskeletal complaints, and fatigue, with no hospitalizations or deaths recorded. In other words, the adverse events were generally mild, similar to those reported in healthy individuals and consistent with findings from other studies evaluating patients with immune-mediated rheumatologic diseases [19,20,21,22,23]. Most studies assessing the reactogenicity of COVID-19 vaccines reported reactogenicity rates close to or exceeding 50%, but with rare occurrences of serious adverse events, corroborating the reactogenicity data observed in our study [15,24,25,26].

Regarding the different vaccine platforms, adverse events were similar in terms of the type of manifestations; however, they were less frequent in patients who received immunization with the inactivated virus vaccine platform (CoronaVac) compared to the other vaccine platforms evaluated in the study (ChadOx-1 and BNT162b2). This finding has also been reported in other prospective cohorts including patients with SLE, where a lower frequency of local symptoms, particularly injection site pain, was associated with individuals who received the inactivated virus vaccine [26,27]. The difference between vaccine platforms regarding the number of adverse events has also been observed in studies involving the general population, where a lower number of adverse events was reported with the inactivated virus platform compared to viral vector and messenger RNA platforms [28].

Concern regarding the impact of vaccines on the risk of disease exacerbation is one of the main reasons for vaccine hesitancy among individuals with immune-mediated diseases, both from healthcare providers and the patients themselves [29]. Our safety analysis regarding SARS-CoV-2 vaccines also evaluated the impact of these immunizations on disease activity and exacerbation in patients with SLE. We identified a significant increase in the proportion of individuals with moderate to high disease activity among patients who received the initial vaccination schedule with ChadOx-1, contrasting with a reduction in the proportion of patients with moderate to high disease activity and an increased remission rate in those vaccinated with CoronaVac. The interpretation of the findings related to disease activity following vaccination should be approached with caution. We observed that a higher proportion of individuals vaccinated with CoronaVac had greater baseline disease activity compared to those who received other vaccine platforms. Therefore, the subsequent reduction in the proportion of patients with moderate-to-high disease activity and the increase in remission rates in this group may reflect, at least in part, the natural course of systemic lupus erythematosus, which is characterized by alternating periods of exacerbation and remission that may not necessarily correlate temporally with immunization and a longitudinal controlled study showed that vaccination did not significantly increase flare rates [30,31]. In contrast, a significant increase in disease activity among individuals vaccinated with ChAdOx-1. This finding may be related to the fact that adenoviral vector-based vaccines are potent inducers of type I interferons, cytokines known to be involved in the pathophysiology of lupus and potentially associated with a higher risk of disease exacerbation. However, the present study was not designed to investigate such mechanisms, as no measurements of cytokines or other inflammatory mediators were performed. Therefore, causal inferences cannot be established from the available data, and the observed association should be interpreted as exploratory [32].

Additionally, we observed variation in the SLEDAI-2K score associated with moderate disease exacerbation in 16.67% of patients after the first dose, 18.44% after the second dose, and 19.05% after the third dose. In contrast, score variation associated with severe flare was identified in fewer than 4% of patients across the different doses administered. The lowest flare rates were observed in the CoronaVac group, whereas the highest rates occurred in the ChAdOx1 group, with BNT162b2 showing intermediate values. Nevertheless, data regarding mRNA vaccines should be interpreted with caution, given the small number of patients who received BNT162b2 in our cohort. The rate of disease exacerbation in patients with SLE has varied widely across studies, ranging from 3% to 20% following SARS-CoV-2 vaccination, and the proportion of flares observed in our study is consistent with that reported in other cohorts [24,33,34]. This variation in disease exacerbation rates across studies may be largely due to heterogeneity in assessment methods and follow-up periods. Our study defined exacerbation based on clinical manifestations observed within 28 days post-vaccination, without limiting exacerbations to cases requiring treatment modification or escalation. This broader definition contrasts with other investigations, such as a prospective cohort that evaluated autoimmune rheumatic disease flares via online questionnaires, where exacerbation was often tied strictly to therapeutic changes [34].

Analyzing this exacerbation rate among the different vaccine platforms, a higher proportion of patients with moderate and severe exacerbation was found among those who received the ChadOx-1 vaccine compared to patients who received the CoronaVac vaccine after the different doses. This increased risk of exacerbation in patients vaccinated with ChadOx-1 was also reported in an international study that included data provided by the COVID-19 Global Rheumatology Alliance [35]. As well as another study, based on data from the European Alliance of Associations for Rheumatology Coronavirus Vaccine Registry, which detected a lower risk of flare in patients with rheumatic disease who received vaccines other than BNT162b2, ChadOx-1 or Moderna [36].

Among the cases of exacerbation, we found greater involvement of the musculoskeletal and renal systems, the latter primarily related to changes in proteinuria, following the pattern of exacerbation manifestations reported in a retrospective study of patients with systemic lupus erythematosus who received the three vaccine platforms evaluated in our study. In the same vein, another multicenter retrospective cohort of patients with SLE reported musculoskeletal domain exacerbation in 31.6% and renal exacerbation in 21% of cases [19,37].

Another relevant aspect analyzed in the present study was the long-term outcome of incident COVID-19 cases. We identified 53 new diagnoses from the start of vaccination up to 12 months after the second dose or the period immediately preceding the administration of the fourth vaccine dose. Most cases occurred in patients who received the initial vaccination schedule with CoronaVac and ChadOx-1, but no statistically significant differences were observed between the vaccine platforms regarding infection cases. The immunogenicity assessment, comparing patients who developed COVID-19 post-vaccination to those who did not during the follow-up period, showed no differences in mean IgG titers and no inferiority in titers among individuals who experienced SARS-CoV-2 infection after vaccination. No deaths or hospitalizations were identified among the incident cases, thereby reinforcing, as reported in other studies, the benefit of vaccination in reducing severe COVID-19 cases in patients with SLE compared to unvaccinated individuals [25,38].

Concerning the limitations of the present study, it is important to highlight some aspects that may influence the interpretation of the results. First, our study was a multicenter effort; however, most participating centers were tertiary referral services specializing in immune-mediated diseases, which may introduce a selection bias by including more complex patients with more severe disease and a higher risk of flare over time. This directly impacts the higher frequency of post-vaccination disease exacerbations observed, especially since we did not have a control group for comparison throughout the follow-up period. In addition, the sample size of patients who received BNT162b2 was relatively small, reflecting the Brazilian vaccination scenario in which CoronaVac and ChAdOx1 predominated, and this should also be considered when interpreting the results.

Furthermore, the observational nature of the study in a real-world design led to the loss of some laboratory data related to immunogenicity and disease activity assessment metrics, especially for patients who received messenger RNA vaccines. The absence of these data may have impacted the analysis of certain outcomes. It should also be noted that a key limitation of our study was the absence of a control group of unvaccinated SLE patients. This limitation reflects the national and international recommendations strongly advising vaccination in this population during the pandemic, which made the inclusion of unvaccinated individuals both unfeasible and ethically inappropriate. Despite these factors, we believe that the results obtained, given the longer follow-up period, multicenter design, and inclusion of a large number of patients with SLE, contribute significantly to the understanding and knowledge of the impact of vaccines in this patient group.

It must be considered that, regarding the impact of vaccination, the COVID-19 pandemic has fostered numerous studies involving immunization in this patient population, which have demonstrated the fundamental role of vaccines in protecting against the development of severe forms of infectious diseases in patients with SLE. Vaccine safety appears to be well established, and concerning the risks of exacerbation, they are reported in studies but do not outweigh the benefits of immunization. The discussion about the optimal timing of vaccination is important due to prior evidence indicating a higher risk of post-vaccination exacerbation in individuals with active disease [39].

## 5. Conclusions

Our study demonstrated that SARS-CoV-2 vaccines are safe for patients with SLE, with adverse events predominantly mild and a low occurrence of severe exacerbations. Lower reactogenicity was observed with the inactivated virus vaccine (CoronaVac), and a higher risk of flare with the viral vector vaccine (ChadOx-1). Thus, our results support the safety of COVID-19 vaccines in patients with SLE and reinforce the importance of immunization, especially in a context of increased risk for severe infections. The choice of an appropriate timing for vaccination, preferably during periods of disease stability, remains an essential strategy to mitigate the risk of disease exacerbation while maximizing the benefits of vaccine protection.

## Figures and Tables

**Table 1:** Frequency of disease activity across different vaccine platforms.

Disease Activity	Total *n*/Total (%)	02 Doses of CoronaVac *n*/Total (%)	02 Doses of ChadOx-1 *n*/Total (%)	02 Doses of BNT162b2 *n*/Total (%)	*p*
Inclusion					**0.026**
Remission	138/360 (38.33)	71/198 (35.86)	53/130 (40.77)	14/32 (43.75)	
Low	118/360 (32.78)	57/198 (28.79)	52/130 (40.00)	9/32 (28.13)	
Moderate to high	104/360 (28.89)	70/198 (35.35)	25/130 (19.23)	9/32 (28.13)	
28 days after 1st dose					**<0.001**
Remission	105/216 (48.61)	70/138 (50.72)	35/77 (45.45)	0/1 (0.00)	
Low	60/216 (27.78)	46/138 (33.33)	13/77 (16.88)	1/1 (100.00)	
Moderate to high	51/216 (23.61)	**22/138 (15.94)**	**29/77 (37.66)**	0/1 (0.00)	
28 days after 2nd dose					0.27
Remission	146/324 (45.06)	76/174 (43.68)	53/119 (44.54)	17/31 (54.84)	
Low	96/324 (29.63)	56/174 (32.18)	30/119 (25.21)	10/31 (32.26)	
Moderate to high	82/324 (25.31)	42/174 (24.14)	36/119 (30.25)	4/31 (12.90)	
Complete vaccination schedule *					
28 days after 3rd dose					0.14
Remission	39/80 (48.80)	19/33 (57.60)	17/32 (53.10)	3/15 (20.00)	
Low	20/80 (25.00)	7/33 (21.20)	8/32 (25.00)	5/15 (33.3)	
Moderate to high	21/80 (26.30)	7/33 (21.20)	7/32 (21.90)	7/15 (46.70)	

**Note:** Frequency of disease activity in patients with systemic lupus erythematosus according to different vaccine platforms (CoronaVac, ChAdOx1, and BNT162b2) at various timepoints: baseline (inclusion), 28 days after the first dose, 28 days after the second dose, and 28 days after the third dose (complete vaccination schedule). Data are presented as absolute numbers and percentages (%), categorized as remission, low disease activity, and moderate to high disease activity. *p*-values refer to comparisons between vaccine platforms at each time point. Statistically significant differences were considered for *p* < 0.05. * Complete vaccination schedule (initial vaccination schedule with 2 doses and a third dose with the BNT162b2 vaccine). Remission (SLEDAI-2K = 0), low activity (SLEDAI-2K 1–5), and moderate to high activity (SLEDAI-2K ≥ 6). Note: The analysis included only patients with a complete assessment of disease activity at the scheduled visit.

**Table 2:** Impact of vaccination on disease activity after the first and second doses, by vaccine platform (paired analysis).

Vaccine	Disease Activity—*n*/Total (%)		*p*
CoronaVac	Inclusion	After 1st dose	
Remission	**46/133 (34.6)**	**68/133 (51.1)**	**0.002**
Low activity	37/133 (27.8)	45/133 (33.8)	0.194
Moderate to high activity	**50/133 (37.6)**	**20/133 (15.0)**	**<0.001**
	**Inclusion**	**After 2nd dose**	
Remission	63/171 (36.8)	74/171 (43.3)	0.152
Low activity	45/171 (26.3)	55/171 (32.2)	0.166
Moderate to high activity	**63/171 (36.8)**	**42/171 (24.6)**	**0.004**
**ChadOx-1**	**Inclusion**	**After 1st dose**	
Remission	37/76 (48.7)	34/76 (44.7)	0.590
Low activity	**28/76 (36.8)**	**13/76 (17.1)**	**0.004**
Moderate to high activity	**11/76 (14.5)**	**29/76 (38.2)**	**0.001**
	**Inclusion**	**After 2nd dose**	
Remission	47/118 (39.8)	52/118 (44.1)	0.446
Low activity	**48/118 (40.7)**	**30/118 (25.4)**	**0.011**
Moderate to high activity	**23/118 (19.5)**	**36/118 (30.5)**	**0.028**
**BNT162b2**	**Inclusion**	**After 2nd dose**	
Remission	14/31 (45.2)	17/31(54.8)	0.366
Low activity	08/31 (25.8)	10/31 (32.3)	0.593
Moderate to high activity	09/31 (29.0)	04/31 (12.9)	0.132

**Note:** The analysis was performed using paired data from patients with complete disease activity assessments before and after each vaccine dose, stratified by vaccine platform (CoronaVac, ChAdOx1, BNT162b2). Data are presented as absolute numbers and percentages of patients in remission, with low disease activity, or with moderate to high disease activity at baseline (inclusion) and after each vaccine dose. *p*-values refer to within-group comparisons between timepoints (pre- vs. post-dose) for each vaccine platform. Differences were considered statistically significant at *p* < 0.05.

**Table 3:** Exacerbation of disease activity based on the SLEDAI score among different vaccine platforms over the follow-up period.

	Total	02 Doses of CoronaVac	02 Doses of ChadOx-1	02 Doses of BNT162b2	*p*
	*n*/Total (%)	*n*/Total (%)	*n*/Total (%)	*n*/Total (%)	
Between T1* and T2**					**<0.001**
Absent	168/210 (80.00)	**121/133 (90.98)**	**46/76 (60.53)**	1/1 (100.00)	
Moderate	35/210 (16.67)	**11/133 (8.27)**	**24/76 (31.58)**	0/1 (0.00)	
Severe	7/210 (3.33)	**1/133 (0.75)**	**6/76 (7.89)**	0/1 (0.00)	
Between T1* and T3***					**<0.001**
Absent	253/320 (79.06)	**143/171 (83.63)**	**81/118 (68.64)**	**29/31 (93.55)**	
Moderate	59/320 (18.44)	**27/171 (15.79)**	**31/118 (26.27)**	**1/31 (3.23)**	
Severe	8/320 (2.50)	**1/171 (0.58)**	**6/118 (5.08)**	**1/31 (3.23)**	
Between T1* and T4****					**0.001**
Absent	168/210 (80.00)	**107/119 (89.92)**	**53/77 (68.83)**	**8/14 (57.14)**	
Moderate	40/210 (19.05)	**12/119 (10.08)**	**22/77 (28.57)**	**6/14 (42.86)**	
Severe	2/210 (0.95)	**0/119 (0.00)**	**2/77 (2.60)**	**0/14 (0.00)**	

**Note:** Exacerbation of disease activity based on the SLEDAI score among different vaccine platforms (CoronaVac, ChAdOx-1, and BNT162b2) over the follow-up period. Proportion of patients with absent, moderate, or severe disease exacerbation at different time points: T1* (baseline visit), T2** (28 days after the first vaccine dose), T3*** (28 days after the second dose), and T4**** (28 days after the third dose—complete vaccination schedule with three doses or heterologous schedule with BNT162b2 as the third dose). Results are presented as absolute numbers and percentages. *p*-values were obtained for comparisons between vaccine groups and were considered statistically significant when < 0.05.

## Data Availability

The data are not publicly available due to privacy restrictions and are only available upon request to the corresponding author at nsartori@hcpa.edu.br.

## Appendix A

**Table A1:** Frequency of adverse events according to vaccine platform.

Adverse Events After 1st Dose	2 Doses of CoronaVac	2 Doses of ChadOx-1	2 Doses of BNT162b2	*p*
	*n*/Total (%)	*n*/Total (%)	*n*/Total (%)	
Local erythema	17/196 (8.67)a	31/128 (24.22)b	3/32 (9.38)a	<0.001
Local hematoma	10/196 (5.10)a	21/128 (16.41)b	4/32 (12.50)ab	0.006
Local swelling	26/196 (13.27)a	48/128 (37.50)b	10/32 (31.25)b	<0.001
Local induration	31/196 (15.82)a	42/128 (32.81)b	10/32 (31.25)ab	0.001
Local pain	89/196 (45.41)a	94/128 (73.44)b	24/32 (75.00)b	<0.001
Nausea and vomiting	40/196 (20.41)ab	37/128 (28.91)b	3/32 (9.38)a	0.043
Fatigue	63/196 (32.14)a	65/128 (50.78)b	10/32 (31.25)a	0.003
Headache	79/196 (40.31)a	77/128 (60.16)b	15/32 (46.88)ab	0.002
Muscle pain	51/196 (26.02)a	61/128 (47.66)b	13/32 (40.63)ab	<0.001
Join pain	59/196 (30.10)a	62/128 (48.44)b	11/32 (34.38)ab	0.004
Fever	17/196 (8.67)a	34/128 (26.56)b	3/32 (9.38)a	<0.001
Dizziness	37/196 (18.88)	33/128 (25.78)	7/32 (21.88)	0.34
**Adverse events after 2nd dose**	**2 doses of CoronaVac**	**2 doses of ChadOx-1**	**2 doses of BNT162b2**	***p***
Local erythema	14/182 (7.69)a	20/118 (16.95)b	3/32 (9.38)ab	0.042
Local hematoma	11/181 (6.08)	9/118 (7.63)	1/32 (3.13)	0.75
Local swelling	23/182 (12.64)	26/118 (22.03)	5/32 (15.63)	0.10
Local induration	19/182 (10.44)a	28/118 (23.73)b	5/32 (15.63)ab	0.008
Local pain	65/182 (35.71)a	63/118 (53.39)b	18/32 (56.25)b	0.004
Nausea and vomiting	27/182 (14.84)	22/118 (18.64)	3/32 (9.38)	0.44
Fatigue	39/182 (21.43)	36/118 (30.51)	11/32 (34.38)	0.11
Headache	56/182 (30.77)	43/118 (36.44)	10/32 (31.25)	0.58
Muscle pain	49/182 (26.92)	36/118 (30.51)	8/32 (25.00)	0.74
Join pain	50/182 (27.47)	38/118 (32.20)	8/32 (25.00)	0.59
Fever	13/182 (7.14)	17/118 (14.41)	1/32 (3.13)	0.058
Dizziness	26/181 (14.36)	22/118 (18.64)	6/32 (18.75)	0.57
**Adverse events after 3rd dose**	**02 doses of CoronaVac +** **BNT162b2** ***n*/total (%)**	**02 doses of ChadOx-1 +** **BNT162b2** ***n*/total (%)**	**03 doses of BNT162b2** ***n*/total (%)**	***p***
Local erythema	11/81 (13.58)	13/72 (18.06)	3/31 (9.68)	0.55
Local hematoma	7/81 (8.64)ab	14/72 (19.44)b	1/31 (3.23)a	0.037
Local swelling	23/81 (28.40)	22/72 (30.56)	8/31 (25.81)	0.88
Local induration	25/81 (30.86)	21/72 (29.17)	7/31 (22.58)	0.68
Local pain	52/81 (64.20)	44/72 (61.11)	16/31 (51.61)	0.47
Nausea and vomiting	14/81 (17.28)	7/72 (9.72)	5/31 (16.13)	0.38
Fatigue	21/81 (25.93)	29/72 (40.28)	6/31 (19.35)	0.053
Headache	34/81 (41.98)	32/72 (44.44)	9/31 (29.03)	0.33
Muscle pain	20/81 (24.69)	26/72 (36.11)	6/31 (19.35)	0.14
Join pain	21/81 (25.93)	28/72 (38.89)	7/31 (22.58)	0.13
Fever	8/81 (9.88)	14/72 (19.44)	1/31 (3.23)	0.056
Dizziness	11/81 (13.58)	15/72 (20.83)	3/31 (9.68)	0.33

**Note:** Frequency of post-vaccination adverse events in patients with systemic lupus erythematosus, stratified by vaccine platform. ab: same letters do not differ according to the *Least Significant Difference* (LSD) test at a 5% significance level. Data are presented as percentages (%). *p* < 0.05 indicates a statistically significant difference.

**Table A2:** Frequency of affected SLEDAI-2K domains among patients who experienced a flare, stratified by flare intensity (moderate or severe).

	Moderate Flare			Severe Flare		
SLEDAI	Inclusion	28 Days After 1st Dose	*p*	Inclusion	28 Days After 1st Dose	*p*
	*n*/Total (%)	*n*/Total (%)		*n*/Total (%)	*n*/Total (%)	
Seizure	0/35 (0.00)	1/35 (2.86)	1.00	0/7 (0.00)	1/7 (14.29)	1.00
Visual disturbance	1/35 (2.86)	4/35 (11.43)	0.36	0/7 (0.00)	3/7 (42.86)	0.19
Psychosis	0/35 (0.00)	1/35 (2.86)	1.00	-	-	-
Vasculitis	0/35 (0.00)	2/35 (5.71)	0.49	0/7 (0.00)	1/7 (14.29)	1.00
Lupus headache	1/35 (2.86)	2/35 (5.71)	1.00	0/7 (0.00)	4/7 (57.14)	0.070
Myositis	0/35 (0.00)	3/35 (8.57)	0.24	0/7 (0.00)	1/7 (14.29)	1.00
Pyuria	0/35 (0.00)	2/35 (5.71)	0.49	0/7 (0.00)	2/7 (28.57)	0.46
Hematuria	0/35 (0.00)	2/35 (5.71)	0.49	0/7 (0.00)	1/7 (14.29)	1.00
Arthritis	3/35 (8.57)	21/35 (60.00)	**<0.001**	0/7 (0.00)	2/7 (28.57)	0.46
Urinary casts	0/35 (0.00)	3/35 (8.57)	0.24	-	-	-
Rash	3/35 (8.57)	6/35 (17.14)	0.48	-	-	-
Mucosal ulcers	1/35 (2.86)	2/35 (5.71)	1.00	-	-	-
Alopecia	4/35 (11.43)	7/35 (20.00)	0.51	1/7 (14.29)	1/7 (14.29)	1.00
Low complement	5/35 (14.29)	5/35 (14.29)	1.00	1/7 (14.29)	1/7 (14.29)	1.00
Increasing DNA binding	5/35 (14.29)	4/35 (11.43)	1.00	0/7 (0.00)	1/7 (14.29)	1.00
Thrombocytopenia	1/35 (2.86)	0/35 (0.00)	1.00	-	-	-
Leukopenia	2/35 (5.71)	6/35 (17.14)	0.26	-	-	-
Proteinuria	1/35 (2.86)	6/35 (17.14)	0.11	0/7 (0.00)	3/7 (42.86)	0.19
**SLEDAI**	**Inclusion**	**28 days after** **2nd dose**	***p***	**Inclusion**	**28 days after** **2nd dose**	***p***
	***n*/total (%)**	***n*/total (%)**		***n*/total (%)**	***n*/total (%)**	
Visual disturbance	0/59 (0.00)	2/59 (3.39)	0.50	0/8 (0.00)	4/8 (50.00)	0.077
CVA	-	-	-	0/8 (0.00)	1/8 (12.50)	1.00
Cranial nerve disorder	-	-	-	1/8 (12.50)	1/8 (12.50)	1.00
Vasculitis	1/59 (1.69)	1/59 (1.69)	1.00	0/8 (0.00)	1/8 (12.50)	1.00
Lupus headache	3/59 (5.08)	14/59 (23.73)	**0.009**	0/8 (0.00)	5/8 (62.50)	**0.026**
Myositis	0/59 (0.00)	1/59 (1.69)	1.00	0/8 (0.00)	1/8 (12.50)	1.00
Pyuria	1/59 (1.69)	14/59 (23.73)	<**0.001**	0/8 (0.00)	2/8 (25.00)	0.47
Hematuria	3/59 (5.08)	10/59 (16.95)	0.078	0/8 (0.00)	1/8 (12.50)	1.00
Arthritis	9/59 (15.25)	26/59 (44.07)	**0.001**	0/8 (0.00)	5/8 (62.50)	**0.026**
Urinary casts	1/59 (1.69)	1/59 (1.69)	1.00	0/8 (0.00)	1/8 (12.50)	1.00
Rash	5/59 (8.47)	13/59 (22.03)	0.073	0/8 (0.00)	5/8 (62.50)	**0.026**
Mucosal ulcers	1/59 (1.69)	7/59 (11.86)	0.061	0/8 (0.00)	1/8 (12.50)	1.00
Pericarditis	0/59 (0.00)	1/59 (1.69)	1.00	-	-	-
Pleurisy	1/59 (1.69)	1/59 (1.69)	1.00	-	-	-
Alopecia	8/59 (13.56)	17/59 (28.81)	0.072	1/8 (12.50)	2/8 (25.00)	1.00
Low complement	7/59 (11.86)	9/59 (15.25)	0.79	0/8 (0.00)	1/8 (12.50)	1.00
Increasing DNA binding	9/59 (15.25)	13/59 (22.03)	0.48	0/8 (0.00)	1/8 (12.50)	1.00
Fever	1/59 (1.69)	1/59 (1.69)	1.00	-	-	-
Thrombocytopenia	3/59 (5.08)	3/59 (5.08)	1.00	1/8 (12.50)	1/8 (12.50)	1.00
Leukopenia	4/59 (6.78)	5/59 (8.47)	1.00	0/8 (0.00)	1/8 (12.50)	1.00
Proteinuria	5/59 (8.47)	15/59 (25.42)	**0.027**	0/8 (0.00)	3/8 (37.50)	0.20
**SLEDAI**	**Inclusion**	**28 days after** **3rd dose**	***p***	**Inclusion**	**28 days after** **3rd dose**	***p***
	***n*/total (%)**	***n*/total (%)**		***n*/total (%)**	***n*/total (%)**	
Seizure	0/40 (0.00)	1/40 (2.50)	1.00	-	-	-
Visual disturbance	0/40 (0.00)	1/40 (2.50)	1.00	0/2 (0.00)	1/2 (50.00)	1.00
CVA	0/40 (0.00)	1/40 (2.50)	1.00	-	-	-
Cranial nerve disorder	0/40 (0.00)	1/40 (2.50)	1.00	-	-	-
Vasculitis	1/40 (2.50)	1/40 (2.50)	1.00	-	-	-
Lupus headache	0/40 (0.00)	6/40 (15.00)	**0.026**	0/2 (0.00)	2/2 (100.00)	0.33
Myositis	0/40 (0.00)	4/40 (10.00)	0.12	-	-	-
Pyuria	1/40 (2.50)	7/40 (17.50)	0.057	-	-	-
Hematuria	1/40 (2.50)	2/40 (5.00)	1.00	-	-	-
Arthritis	2/40 (5.00)	21/40 (52.50)	**<0.001**	0/2 (0.00)	1/2 (50.00)	1.00
Rash	1/40 (2.50)	5/40 (12.50)	0.20	0/2 (0.00)	1/2 (50.00)	1.00
Mucosal ulcers	0/40 (0.00)	1/40 (2.50)	1.00	-	-	-
Pleurisy	1/40 (2.50)	0/40 (0.00)	1.00	-	-	-
Alopecia	2/40 (5.00)	8/40 (20.00)	0.091	-	-	-
Low complement	2/40 (5.00)	4/40 (10.00)	0.68	1/2 (50.00)	1/2 (50.00)	1.00
Increasing DNA binding	2/40 (5.00)	5/40 (12.50)	0.43	1/2 (50.00)	1/2 (50.00)	1.00
Fever	0/40 (0.00)	2/40 (5.00)	0.49	-	-	-
Leukopenia	0/40 (0.00)	2/40 (5.00)	0.49	-	-	-
Proteinuria	1/40 (2.50)	6/40 (15.00)	0.11	-	-	-

**Note:** Data are expressed as the number and percentage of patients with each affected domain at inclusion and 28 days after each vaccine dose. *p*-values indicate statistically significant differences (*p* < 0.05) between the evaluated time points. CVA; Cerebrovascular Accident.
